# What’s more general than a whole population?

**DOI:** 10.1186/s12982-015-0029-4

**Published:** 2015-08-25

**Authors:** Neal Alexander

**Affiliations:** MRC Tropical Epidemiology Group, London School of Hygiene and Tropical Medicine, Keppel Street, London, UK

## Abstract

Statistical inference is commonly said to be inapplicable to complete population studies, such as censuses, due to the absence of sampling variability. Nevertheless, in recent years, studies of whole populations, e.g., all cases of a certain cancer in a given country, have become more common, and often report *p* values and confidence intervals regardless of such concerns. With reference to the social science literature, the current paper explores the circumstances under which statistical inference can be meaningful for such studies. It concludes that its use implicitly requires a target population which is wider than the whole population studied — for example future cases, or a supranational geographic region — and that the validity of such statistical analysis depends on the generalizability of the whole to the target population.

*If Czech history could be repeated, we should of course find it desirable to test the other possibility each time and compare the results. Without such an experiment, all considerations of this kind remain a game of hypotheses* [1].

## Introduction

**Fig. 1:**
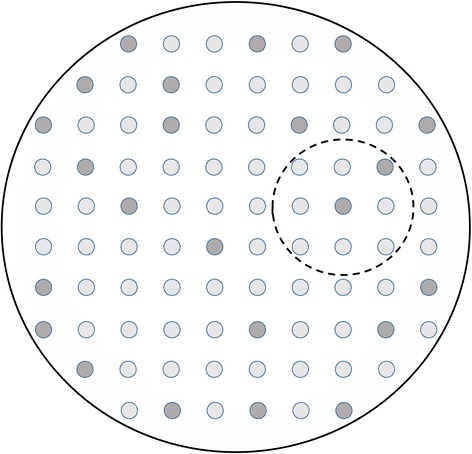
The classical situation of a sample (small dashed circle) drawn from a population (large solid circle). The darker shading represents units with a characteristic of interest

Classical frequentist statistics relies on the notion of a sampling population to justify probability statements such as *p* values or confidence interval coverage. A sampling population is a source of variation over putative repeated samples, as illustrated in Fig. 1. For such variation to occur, the sample must be smaller than the population, otherwise ‘sampling errors disappear altogether’ [2] (p659) and *p* values tend to zero. This is formalized in the finite population adjustment, which reduces standard errors towards zero as the size of the sample approaches that of the population [3] (p436). This adjustment is rarely used because, in the classical framework, the sample is much smaller than the population. Sometimes this inequality is simply asserted — e.g., ‘We cannot study all the population’ [4]. However, advances in computerization of health information, for example through national cancer registries [5] and mass genotyping [6], have made it more feasible to study groups which can reasonably be called ‘whole populations’. Although missing data can rarely, if ever, be ruled out, some studies, for example based on cancer registries, have achieved very low levels [7, 8]. The social sciences recognise repeated sampling to be inapplicable to certain kinds of whole population studies [9]. For example, when studying characteristics of the ten largest cities in a given country, based on data aggregated from the latest national census, re-doing the study would not subject the data to sampling variation. To understand how statistical inferential might, nevertheless, be applicable to whole population studies, we need to distinguish different uses of the word ‘population’.

**Fig. 2:**
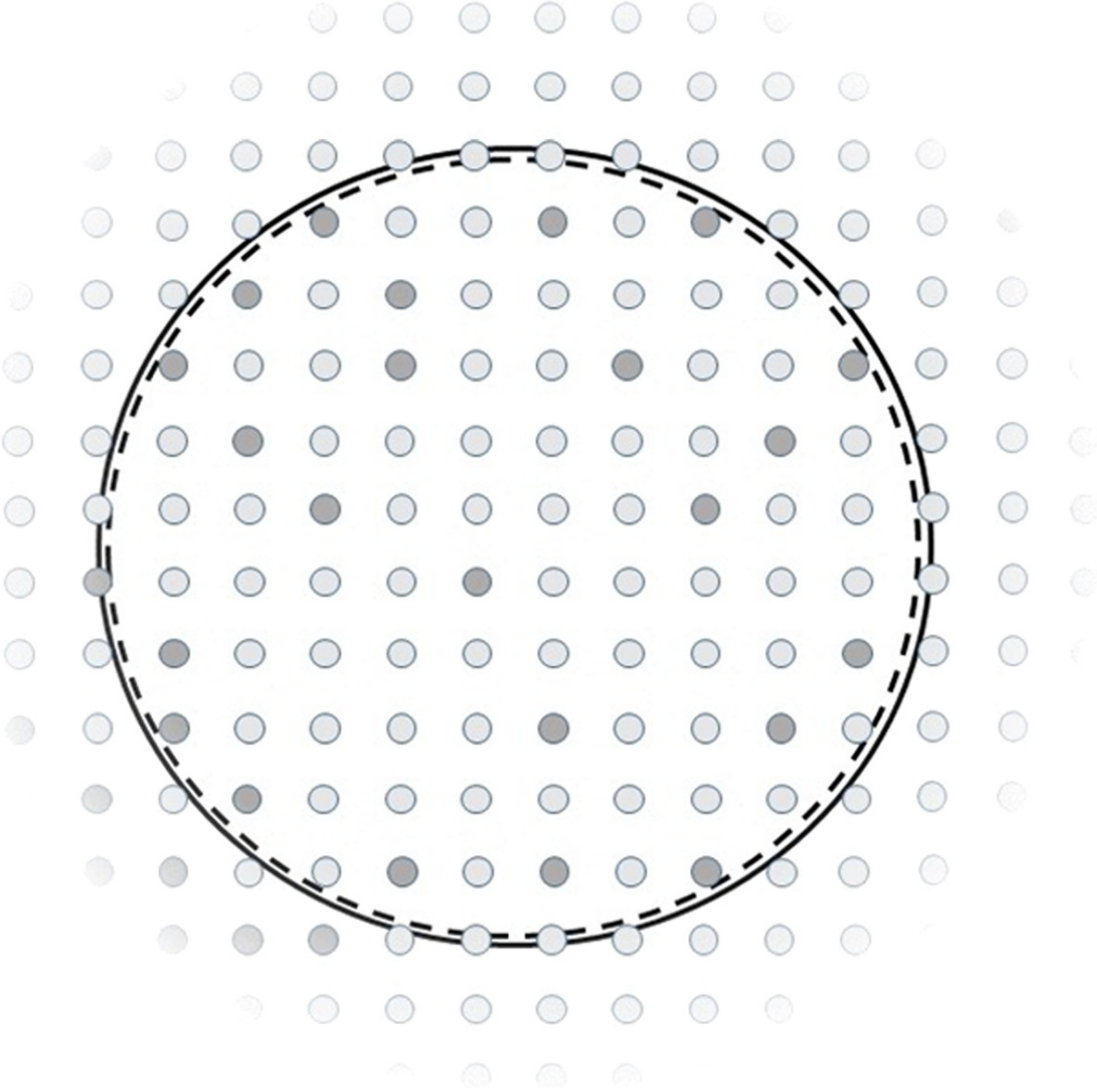
In a whole population study, the sample (dashed circle) has become so large as to coincide with the population (solid circle). The use of confidence intervals, *p* values, or similar probability statements implies a claim to generalizability to some group beyond the study population: namely the target population, represented by the area outside the two circles. As in Fig. 1, presence or absence of a characteristic is indicated by darker or lighter shading. Authors of whole population studies often do not try to delimit their generalizability. In the figure, such indeterminacy is represented by the target population fading away from the original population: we may not know to what spatial or temporal range the findings may be applicable. Similarly, we may not be able to judge whether the prevalence of the characteristic remains similar in the target population, rather than increasing or decreasing (as it does to the bottom left and top right, respectively, of the figure)

One way to preserve sampling-based statistical inference is to adduce a group that is even more general than the whole population that was studied. This kind of wider group is called here a target population, although the term is sometimes used as a synonym for the sampling population [10]. The target population is the group ‘about which conclusions are to be made’ [11] but, when it differs from the sampling population, it is not subject to the sampling variation formalism. Some authors define target populations to be real [12] (p361) while for others they do not necessarily exist, at least not yet. For example, Kirkwood and Sterne say that enumeration of a target population may be impossible because it ‘often includes not only all persons living at present but also those that may be alive at some time in the future’ [13] (p10). Despite being currently intangible, such target populations can be important to the application in hand, the cited authors’ example being future recipients of an experimental vaccine. Target populations (in this sense) are sometimes called superpopulations, which comprise ‘all possible persons that ever were or ever could be targets of inference’ [12].

In the following sections we look at attempts to retain statistical inference in whole population studies, then argue that the problem is better seen as one of generalizability.

### Population-free statistics?

Attempts have been made to justify statistical inference to whole populations by resorting to alternative techniques, in particular the bootstrap, and Bayesian estimation [9, 14].

The bootstrap generates variation by sampling the original data with replacement. This can actually be carried out in practice, unlike the notional repeated sampling of a population. However the validity of the bootstrap depends on ‘independent identical sampling from an unknown distribution’ [15] so the original data are themselves still assumed to result from a sampling process.

Bayesian inference is based on *a priori* probability distributions (‘priors’) and a likelihood model for data and parameters. The meaning of the prior probabilities does not rely on sampling properties of populations. By applying Bayes’ theorem to the model and available data, inference is made about the parameters, for example in the form of credible intervals, i.e., percentile ranges calculated from the parameters’ posterior distributions. If we already have complete data on the whole population, then statements about probabilities must refer to something wider. Since we have complete data on the population why do summary statistics not suffice, without confidence or credible intervals? For some situations summary statistics *will* suffice (see Table 1). If they do not, it is because we are interested in a wider group than the original ‘whole’ population, namely the target population. At the technical level, Bayesian analysis can be run without giving any thought to this target population but this does not mean that the results are validly applicable to it. Moreover, many Bayesian problems allow ‘matching’ priors which replicate frequentist results [16]. It would seem vacuous to consider one approach valid and the other invalid when they can be designed to give the same results. Similarly, inference, whether Bayesian or not, may also be expressed in terms of parameters of a hypothesised data generating mechanism, often expressed as a probability model. This can be done even when the data comprise a whole population but if the parameters are not estimated perfectly then more could be learned from further data, i.e., from the target population, assuming the same data generating mechanism applies.

**Table 1:** Consider the following two examples of a binary outcome with complete population coverage

*Presidential election:* there is 100% turnout in a national presidential election, with each vote being either for candidate A or candidate B.
*Cancer registry:* male or female sex is registered for all cases in a national registry which has 100% coverage of the type of cancer in question.
In the election example, is it meaningful to estimate a sampling error for the proportion voting for candidate A? The answer seems to be clearly ‘no’. This is because the purpose of the election is to choose a president, which is done on the basis of the observed proportion of votes cast. Any kind of interval estimate serves no purpose because there is no generalizability beyond the election.
In the cancer registry example, is it meaningful to estimate a sampling error for the proportion of cases who are female? Some would say ‘no’ on the basis that it’s a complete population enumeration with no sampling error. Similar examples in the literature show that some authors would say ‘yes’. This implies an attempt to generalize beyond the population observed, but what is this wider target population? Conceivably future cases, or a wider, supranational geographical area, although often this is left unspecified.

### Representativeness and generalizability

The above reasoning suggests that the validity of either frequentist or Bayesian inference depends on the extent to which the group being studied — whether or not it is a whole population — is representative of the target population. Porta [10] allows a sample to be representative of a population if it is ‘typical in respect of certain characteristics, however chosen’, i.e., without requiring a particular selection method such as random sampling. Hence a study group can potentially be representative of a given target population, and its findings generalizable to it, even if the target population is not amenable to sampling.

The current paper argues that the problem of inferential methods in whole populations is most usefully understood as one of generalizability (Fig. 2). Thinking in these terms helps show that inferential methods are meaningless for some situations while for others their use is at least arguable (see Table 1).

Lack of information on generalizability has been identified as a limiting factor in the translation of research findings to policy [17–19]. The CONSORT guidelines for clinical trials [20], and STROBE for observational studies [21] mandate discussion of generalizability but give no guidance on how to assess it. Point 21 of STROBE, for example, is ‘Discuss the generalisability (external validity) of the study results’. Several frameworks for assessing generalizability have been proposed [17, 22–24]. Inferential analysis of whole population studies is justified only as far as such studies are generalizable, yet reporting of generalizability in other kinds of study is often poor [18, 19]. The extent to which this also applies to whole population studies is briefly assessed in the following section.

### Literature review on generalizability of whole population studies

A literature review was carried out to assess the extent to which whole population studies assess their own generalizability. The search term ‘whole population [TI]’ was used in PubMed on 25 February 2015, restricted to publications in 1994 or later, yielding 64 publications. They were retained if containing primary data on studies of humans, and reporting *p* values or confidence intervals in the abstract. The full text of each of the resulting 13 studies was reviewed for description of its generalizability or external validity (Table 2). The populations were typically either whole countries (e.g., Iceland) or subnational administrative regions of different sizes (e.g., Western Australia, Isle of Wight). Only two papers [25, 26] made reference to wider target populations to which the results might be generalized, and which might give meaning to the *p* values and/or confidence intervals used. These findings can only be suggestive because a limitation of the search is that it does not cover all studies which the current paper would call ‘whole population’. For example, the previously-cited Danish National Acute Leukemia Registry [7] has estimated coverage of more than 98 %, and a PubMed search for its name yields 11 studies, but none of these met the current search criteria. Among the studies which *were* identified, however, few are concerned about their generalizability despite the fact that, on the reasoning of the current paper, the validity of their statistical inference depends on it. In particular none of them mentioned the STROBE guidelines which mandate that generalisability be considered.

**Table 2:** ᅟ

Publication Year	Reference Number	Population	Assessment of generalizability or external validity
2015	[26]	Western Australia	‘We are confident about the generalisability of our analytic findings on associations with stimulant medication use Australia-wide’
2014	[33]	Western Australia	none
2014	[25]	North East Scotland	‘Our results could be generalisable to young children across the UK'
2014	[34]	Western Australia	none
2013	[35]	Western Australia	none
2012	[36]	Western Australia	none
2012	[37]	Western Australia	none
2012	[38]	Iceland	none
2011	[39]	Western Australia	none
2010	[40]	Scotland	none
2004	[41]	‘Top End’ of the Northern Territory of Australia	none
2001	[42]	Isle of Wight, United Kingdom	none
1998	[43]	Isle of Wight, United Kingdom	none

## Conclusions

Whole population studies often calculate *p* values and confidence intervals which have no explicit theoretical basis. This is a problem that social sciences have, perhaps, confronted more directly than epidemiology [9, 27]. Having concluded that purely statistical workarounds are futile, should we eschew inferential statistics altogether and concentrate instead on descriptive statistics [28]? The current paper advocates a less absolute position; that the conclusions of whole population studies are valid to the extent that their study populations are representative of a wider target population. Hence the problem is converted into one of generalizability. In turn this should be manifest in each study’s research question. For example, who is intended to benefit from analysis of national cancer registry data to be applied; future cases in the same country, and/or those further afield? If the former, making this explicit may highlight needs for particular statistical methods, such as time series analysis on the existing data disaggregated by time. Some target populations may not be possible to sample, e.g., because they lie in the future. Similarly, causal inference deals with unobserved or counterfactual outcomes [29, 30], and can be cast in terms of target and source populations [31], and is a promising approach for analysis of whole population studies.

In epidemiology, although aspirations to generalizability are not always met, groundwork has been done on its objective assessment [17, 22–24, 32]. For inferential statistics of whole population studies to be more meaningful, they should fully comply with existing guidelines on reporting generalizability [20, 21], and these guidelines should themselves be updated to reflect the developing best practice.
